# Primary Transverse Closure Compared to Open Wound Treatment for Primary Pilonidal Sinus Disease in Children

**DOI:** 10.3390/children7100187

**Published:** 2020-10-17

**Authors:** Michèle Pfammatter, Tobias E. Erlanger, Johannes Mayr

**Affiliations:** 1 Department of Pediatric Surgery, University Children’s Hospital Basel, 4031 Basel, Switzerland; michele.pfammatter@ukbb.ch; 2 Department of Clinical Research, University of Basel, University Hospital Basel, 4031 Basel, Switzerland; tobias.erlanger@usb.ch

**Keywords:** child, pilonidal sinus disease, recurrence, economics, LOS, primary transverse closure, surgical excision

## Abstract

We aimed to compare the outcome of two different operative methods to correct pilonidal sinus disease (PSD) in children, i.e., excision and open wound care (OW) versus excision and primary transverse closure (PC) of the wound. In this retrospective, observational study, we extracted data from the medical records of 56 patients who underwent surgery for PSD at our institution between 1 January 2006 and 31 December 2016. To test whether the primary variable, i.e., rate of PSD recurrence, differed between the two surgical groups, a logistic regression model was fitted. Secondary explanatory variables were total length of stay (LOS) at the hospital, complications, sex and age of patients, seniority of the surgeon in charge, and volume of excised specimen. Overall, 32 (57%) children and young adults underwent OW, while 24 (43%) patients were treated by PC. Mean age at operation was 15.5 years in either group. PSD recurred in 12 of 32 (37.5%) children in the OW group and in 3 of 24 (12.5%) children in the PC group (ratio: 0.19, 95% confidence interval [95% CI] 0.03–1.07). Thus, treatment of primary PSD by PC proved superior with respect to PSD recurrence. Moreover, our study did not bring to light any high-grade complications in the PC group, and postoperative pain was minimal. Less invasive treatment approaches for chronic PSD are typically performed in an outpatient setting and offer reduced morbidity, low rates of PSD recurrence, and shortened periods of time to return to work or social activities. More radical operations of PSD should be reserved for recurrent PSD where less invasive approaches have failed several times.

## 1. Introduction

Although the exact pathomechanism of pilonidal sinus disease (PSD) has not been conclusively established, ingrown hair between the buttocks is considered to be the main cause of PSD [1]. The German S3 guideline (“Leitlinie Sinus pilonidalis”) and other papers described broken pieces of hair which drill into the subcutaneous tissue by mechanical friction supported by dandruff, which is thought to act as small barbs [2]. Doll et al. demonstrated that the hair found in the sinus originated from the head and entered the natal cleft after haircuts [3]. Therefore, risk factors are the quality of hair (strong and inflexible hair), sedentary occupation, obesity, repetitive trauma or irritation at the natal cleft, hirsute body habitus, and perspiration [4,5]. 

PSD affects 1.2 children per 1000 children at a mean age of 15 years (range: 12 to 19 years), but the frequency seems to have increased in recent decades [2,6]. In the United States, PSD affects approximately 70,000 persons per year [5]. The disease occurs most frequently in the second or third decade of life but also appears in adolescents and children, predominantly in male adolescents [2,7]. PSD is considered an acquired disease [8]. 

To prevent chronic or acute infections, surgical excision of the sinus and its fistulas represent the most common intervention [1,6,8,9,10,11,12,13,14,15,16,17]. Nevertheless, no gold standard for the treatment for PSD has emerged so far [1,2,18,19]. 

The various treatment options can be categorized into those involving open wound treatment, those applying various forms of primary wound closure, and those involving newer, minimally invasive interventions [4,19,20]. Open wound treatments are associated with a low recurrence rate but are cumbersome. In addition, it sometimes takes years until secondary wound healing is completed [1,5,19]. The most reliable predictors for recurrence of PSD are the type of surgical method, length of follow-up interval, and intra-operative use of methylene blue [21,22,23]. 

Elective surgical procedures result in lower recurrence rates compared to emergency surgeries. In addition, absence of a history of previous surgery is associated with a lower recurrence rate when compared to repeated surgery [24]. However, in a Cochrane meta-analysis of randomized clinical trials (RCTs) focusing on PSD treatment in patients aged >14 years comparing primary healing to secondary healing, authors noted that only the management of acute abscesses by incision and drainage is unequivocally established [1].

The influence of high body mass index (BMI) and surgical site infection (SSI) on the rate of PSD recurrence is discussed controversially [8,21,22,25,26,27,28]. While topical gentamycin application lowers the rate of SSI, it does not improve the long-term rate of PSD recurrence [21].

The cohort of patients undergoing primary wound closure should be subdivided into patients treated by midline or off-midline suture techniques [4,10,11,14]. Although midline closure results in a shorter healing time than open wound methods, this approach is associated with a higher recurrence rate [4,9,11,14,20]. Several off-midline suture techniques and reconstructive operations to correct the deep and hairy natal cleft have been published [1,10,11,17,18]. 

**Figure 1:**
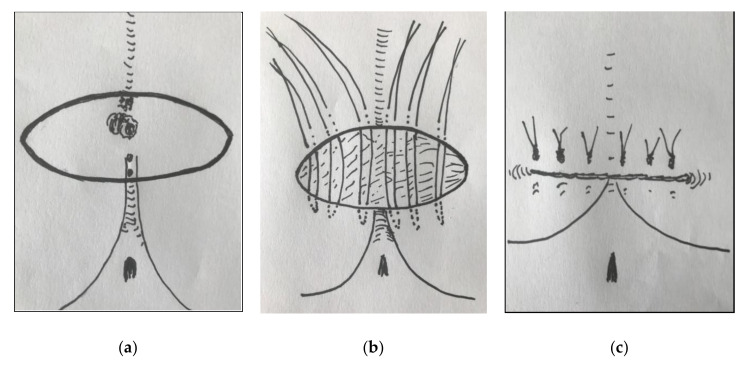
Surgical procedure. (**a**) The planned line of elliptic excision contains orifices of sinus tracts; (**b**) after excision of the sinus tracts, hair and debris are removed, followed by curettage of the wound. Allgöwer stitches are placed outside the midline; (**c**) tying the knots closes the wound and flattens the natal cleft.

Since 2015, we have been using another method of primary closure termed primary transverse closure. After transverse elliptic excision, the margins of the wound are merged horizontally; thus, only a small section of the scar crosses the midline (Figure 1). This surgical technique does not only remove the sinus but also flattens the natal cleft.

In the absence of standardized surgical management of PSD and the paucity of literature on the treatment of PSD in children, investigations on the outcome of different surgical treatment options are of importance [29,30].

This study aimed to establish whether primary transverse closure is superior to open wound care with regard to the rate of PSD recurrence, total LOS, and complication rate. 

## 2. Patients and Methods

### 2.1. Study Design

We undertook a retrospective, observational, single-center study, in accordance with ethical guidelines defined by the Declaration of Helsinki. Ethical approval was obtained from the Ethics Committee of Northwest and Central Switzerland (EKNZ; ID 2017-01098). 

The primary endpoint of the study was the rate of PSD recurrence after surgery. Secondary endpoints included total length of stay (LOS) at the hospital, postoperative complications including bleeding, infection, and wound healing problems, and duration of absence from school or work. The secondary variables were analyzed descriptively. 

We retrieved the medical records of patients who underwent surgery for PSD at our institution between 1 January 2006 and 31 December 2016. During the study interval, we changed our surgical technique to manage primary PSD from total excision of PSD followed by open wound care to elliptical excision of PSD and primary transverse wound closure. In the first 5-year period, all patients underwent total excision and open wound care with or without negative pressure treatment. Thereafter, the surgeons in charge offered transverse elliptic excision and primary transverse closure of the wound to all patients in whom primary closure of the wound seemed feasible. 

The surgeons in charge were obliged to verbally describe the surgical procedures, and drawings were used to explain the advantages and disadvantages of the surgical methods to the patients and their parents. This took place several days before the surgical intervention. One day before the scheduled operation, patients and their parents were seen again to address any remaining questions. Selection of patients for the two procedures was thus based on the preference expressed by families after having been informed by the surgeon in charge.

We allocated all eligible patients to the open wound treatment group (OW) or primary transverse closure group (PC). Information extracted from the medical records was entered into an anonymized electronic database.

### 2.2. Inclusion and Exclusion Criteria

Patients who had undergone either OW (with or without postoperative VAC^®^ treatment) [19] or PC surgeries had to be between 8 and 18 years of age at the time of surgery. 

We excluded patients who had undergone prior excisional treatment of PSD as well as those who had been managed by surgical techniques other than PC or OW. 

### 2.3. Study Procedures

We recorded the patients’ age, sex, percentile of their body weight at the time of surgery, chosen treatment (PC or OW), position of the surgeon in charge (attending pediatric surgeon, pediatric surgical resident), maximum volume of the excised specimen, duration of postoperative VAC^®^ therapy, and number of VAC^®^ changes needed. Using a visual analog scale (VAS), we assessed the pain perceived by the children on the day of operation as well as on the subsequent postoperative days 1 to 6. In addition, we recorded the number of postoperative laser treatments applied for relapse prevention, time to last follow-up consultation, total number of surgeries, and number of additional anesthetic procedures. 

All operations in children and adolescents were performed under general anesthesia. We employed similar surgical techniques in all patients. The surgeons in charge were either an attending pediatric surgeon or pediatric surgical resident supervised by an attending pediatric surgeon. 

### 2.4. Primary Endpoint

The primary endpoint in this investigation was the PSD recurrence rate. Recurrence was defined as a persistent putrid secretion from the wound as well as clinical or histological evidence of pilonidal sinus ≥1 month after the primary excision [16,19] with an indication for revision operation. No further intervention was necessary if a plain scar or small asymptomatic pore had resulted from the surgical intervention.

### 2.5. Secondary Endpoints

Secondary endpoints were LOS, postoperative complications, and duration of absence from school or work. Total LOS was defined as the overall period spent in hospital due to PSD. This included the inpatient stay as well as outpatient consultations. All inpatient days and stays at the day clinic counted as full days. Visits to the outpatient clinic and emergency department counted as half days, based on estimated preparation time, time for traveling to the clinic, consultation, and the time needed to obtain medications from the pharmacy. 

Postoperative complications included problems with VAC^®^ wound dressing (KCI Inc.^®^, San Antonio, TX, USA), and other complications during inpatient stay. Specifically, we recorded bleeding from the wound, SSIs, wound healing problems, and newly diagnosed allergies. If PSD reoccurred subsequently, the prior complications were regarded as forerunners of PSD relapse and were not counted as complications. We categorized the severity of complications according to the Clavien–Dindo classification of surgical complications [29]. 

Duration of wound healing in the OW group encompassed the timespan between excision and completed wound healing. In the PC group, time to wound healing was the period until suture removal if no wound healing problems had occurred. If wound healing was not completed at this time point, it was handled in the same way as in the OW group. 

To quantitate the absence from school or work, we categorized the periods in 2-week intervals, ranging from 0 to 2 weeks (category A) up to >14 weeks (category H).

### 2.6. Statistical Analyses

To test whether there was a difference in the rate of PSD recurrence between the two surgical groups, a multivariable logistic regression model was fitted. The outcome variable was PSD recurrence (yes/no), and explanatory variables were type of surgical treatment, sex, age, seniority of the surgeon, and volume of excised specimen. Missing data were imputed using multiple imputations with chained equations. All variables available were used for imputation. It was assumed that missing data occurred completely at random. 

Secondary endpoints were analyzed descriptively. Summary statistics stratified by surgical method were computed for all patients. For numerical variables with normally distributed data, means and standard deviation (SD) were calculated. For non-normally distributed data, median and interquartile range (IQR) were computed. For categorical data, frequencies were tabulated.

We analyzed the data using R language and environment (https://www.R-project.org/). For imputation and the logistic regression model, the “mice” package was used. 

## 3. Results

### 3.1. Patient Demographics and Surgical Details

We included 56 patients in our analysis, i.e., 32 (57.1%) patients in the OW group and 24 (42.9%) patients in PC group. Datasets were complete for all patients except for two (3.6%) patients for whom the volume of excised specimen was not recorded. For these two missing values, we applied multiple imputations. In both groups, more patients were male (OW 19 (59.4%) patients and PC 17 (70.8%) patients). 

Overall, 17 of 32 (53.1%) patients in the OW group and eight of 24 (33.3%) patients in the PC group had undergone previous incision of acute pilonidal abscess. Mean (SD) age at operation was 15.5 years (1.0 years) in the OW group and 15.5 years (1.4 years) in the PC group. Median body weight of patients was 73 kg (IQR: 63.8, 85.0 kg) in the OW group and 66.3 kg (IQR: 57.3, 77.0 kg) in the PC group. Overall, 50.0% of patients in the OW group and 29.2% of patients in the PC group had a body weight exceeding the 97th percentile (Table 1).

One of six attending pediatric surgeons performed the operation in 11 of 32 (34.4%) patients in the OW group and in 12 of 24 (50.0%) patients in the PC group. 

Mean (SD) volume of removed tissue was 28.7 cm^3^ (37.9 cm^3^) in the OW group and 19.2 cm^3^ (22.0 cm^3^) in the PC group. 

We applied VAC^®^ wound dressing in 26 of 32 (81.2%) patients in the OW group. The number of VAC^®^ changes ranged from 0 (one patient) to 14 (one patient) in the OW group. In the PC group, a suction drain was left in place for a median of 2 days (IQR: 0, 0 days) in 17 of 24 (70.8%) patients (Table 1).

Median duration of follow-up was 15.9 months (IQR: 4.5, 31.5 months) in the OW group and 6.3 months (IQR: 2.6, 19.1 months) in the PC group. 

### 3.2. Rate of PSD Recurrence (Primary Endpoint)

In the OW group, 37.5% of patients suffered PSD recurrence, while in the PC group, only 12.5% of patients experienced PDS recurrence. The unadjusted ratio of the odds of recurrence after PC and OW was 0.24. When considering potential confounders in the logistic regression model, the ratio decreased to 0.19 with a 95% confidence interval (95% CI) of 0.03–1.07. Odds ratios (95% CI) of explanatory variables were as follows: sex 1.18 (0.25–5.49), age 0.6 (0.31–1.18), seniority of surgeon 2.29 (0.47–11.24), and volume of excised specimen 2.29 (0.47–11.24).

### 3.3. Total Length of Hospital Stay (LOS)

Median total LOS amounted to 15.8 days (IQR: 10.2, 25.5 days) for the OW group and 9.0 days (IQR: 7.5, 11.5 days) for the PC group (Table 2). LOS in the pediatric surgical inpatient ward was similar in the two groups (median 6.5 days (IQR: 3.8, 12.0 days) in the OW group and 6.0 days (IQR: 5.0, 7.0 days) in the PC group). Median outpatient LOS was 3.0 days (IQR: 2.0, 4.8 days) in the PC group, compared to 8.5 days (IQR: 5.5, 14.5 days) in the OW group (Table 2). 

In our study, LOS did not appear to depend on the seniority of the surgeon in charge of the operation (pediatric surgical resident vs. attending pediatric surgeon; Table 1). In line with our findings, Iesalnieks et al. reported that the seniority of the surgeon was not associated with PSD recurrence after surgery [14]. 

### 3.4. Absence from School or Work

Due to missing data, overall duration of absence from school or work was analyzed for only 12 (37.5%) patients in the OW group and 12 (50.0%) patients in the PC group. Overall, seven (58.3%) patients in the PC group and four (33.3%) patients in the OW group returned to school or work within 2 to 4 weeks (Table 2). 

### 3.5. Complications and Postoperative Pain

Complications occurred in 19 of 32 (59.4%) patients in the OW group and in 13 of 24 (54.2%) patients in the PC group. Complications were grouped according to the Clavien–Dindo classification of surgical complications [29] (Table 3). 

Pain was rated on the day of operation as well as on days 1 to 6 after the operations. Patients in the PC group rated the pain on the day of operation slightly higher than those in the OW group. However, mean VAS sores on all subsequent days up to day 6 were somewhat lower in the PC group than the OW group. The highest score (3.2) was measured on day 5 in the OW group. Patients in the PC group were free of pain from day 5 onwards (Table 4).

### 3.6. Number of Operations and Additional Anesthesia 

In the OW group, seven (21.9%) patients underwent two surgeries, while three (9.4%) patients required three operations, including the initial excision. One patient required four operations, and another patient underwent five operations. In the PC group, one patient each underwent two, three, and five surgeries in total, including the initial excision (Table 5).

No additional anesthesia was required in the PC group whereas five (15.6%) patients in the OW group were exposed to one additional anesthesia, and two (6.2%) patients underwent three additional anesthetic procedures (Table 5).

### 3.7. Postoperative Laser Hair Epilation 

In both groups, 50.0% of patients (i.e., 16 patients in the OW group and 12 patients in the PC group) underwent postoperative laser hair epilation treatment to prevent PSD recurrence, whereby the number of laser epilation sessions differed among patients (Table 5).

## 4. Discussion

### 4.1. Rate of PSD Recurrence 

Although the difference in PSD recurrence rate between the OW and PC groups did not reach statistical significance, a markedly lower number of patients experienced PSD recurrence in the PC group (PC 12.5% vs. OW 37.5%). Braungart et al. reported a recurrence rate of 22% after pilonidal sinus excision and primary midline closure (PMC) with flattening of the natal cleft [18]. Iesalnieks et al., who treated the majority of PSD patients by excision and PMC, observed a recurrence rate of 25% after primary surgery for PSD and of 48% after surgical treatment of recurrent PSD [14]. Dogan et al. proposed the inverse “D” incision technique with lateral placement of the suture line. These authors reported postoperative wound healing complications in 6.3% of patients, recurrence rate of 1.3%, and a mean LOS of 2.4 days. The mean follow-up interval was 36 months [10]. 

Flattening the natal cleft by a cleft lift procedure to treat refractory PSD has been proposed by Bascom and Bascom [30]. Søndenaa et al. concluded that the Limberg flap helps to flatten the natal cleft, thereby reducing hair accumulation and lowering the rate of PSD recurrence [5].

In an RCT with a 7-year follow-up comparing midline, unshifted adipofascial turnover flap, off-midline shifted adipofascial turnover flap (inverse “D”-shaped flap), and Karydakis flap, Caliskan et al. detected no significant differences between groups with respect to PSD recurrence and postoperative complications [11]. 

A multicenter study from Switzerland and Austria published in 2020 reported a rate of PSD recurrence of 20% [31]. In our investigation, PSD recurred in 12 of 32 (37.5%) patients in the OW group and in three of 24 (12.5%) patients in the PC group. Hatch et al. reported results of a series of 235 PSD patients treated by the Bascom cleft lift technique [32]. Despite a high rate (54%) of minor and major complications, they reported a favorable recurrence rate of 4.7% [32]. 

In a 5-year follow-up study, Fahrni et al. reported lower recurrence rates for wide local excision with secondary wound healing (recurrence rate: 11.3%) when compared to limited excision (recurrence rate: 27.6%) or wide excision with primary wound closure (recurrence rate: 26.8%) [23]. The American Society of Colon and Rectal Surgeons (ASCRS) and a meta-analysis from Switzerland strongly recommend flap-based procedures in the setting of complex and recurrent PSD to reduce the risk of recurrence [4,33].

When comparing the effectiveness of the various therapeutic options, the influence of the duration of follow-up monitoring on the rate of PSD recurrence must be kept in mind [34]. Primary asymmetric excision and scar lateralization in PSD result in the lowest recurrence rates, whereas placing the scar in the midline is associated with high recurrence rates [34].

### 4.2. Total Length of Hospital Stay

Our study showed a significant reduction in total LOS when transverse elliptical excision and PC rather than OW were applied (9.0 days versus 15.8 days, respectively; hazard ratio: 1.93; CI: 3.52–1.06; *p* = 0.031). This was caused mainly by the shorter duration of overall outpatient stay, while inpatient LOS for the PC and OW groups was comparable (6.0 vs. 6.5 days, respectively).

Other authors reported a shorter mean LOS of 2.7 days (range: 1 to 14 days) for excision and midline closure and even for outpatient treatment, e.g., for Gips procedure or application of crystallized phenol in the treatment of PSD [9,35,36].

The length of inpatient stay in the OW group resulted from our procedure to change the first dressing at the hospital to monitor early complications and train patients and their parents in the handling of VAC^®^ and pain management. In the PC group, the patients were also retained in hospital for a few days to ensure that there were no early complications or problems with pain management. In addition, we considered hospitalization advisable for children and adolescents because this reduced their activity level and allowed them to spend more time in the prone or lateral position in order to protect the wound from external disruptive forces. With growing experience with PC, the inpatient stay might be shortened, with the further treatment scheduled in the outpatient clinic. Shorter LOS is regarded as a gain for patients as well as their families because staying in hospital might be cumbersome.

### 4.3. Volume of Excised Tissue

We calculated a mean volume of excised tissue of 28.7 cm^3^ (SD: 37.9 cm^3^) for the OW group and 19.2 cm^3^ (SD: 22.0 cm^3^) for the PC group. While there was no significant difference in the volume of excised tissue between the two groups, the extent of excised tissue correlated with total LOS, which was longer in the OW group than the PC group. Caliskan et al. reported the amount of excised tissue in terms of median diameter of the pilonidal cyst (20.1 mm; SD: 8.0 mm) rather than the volume, which makes comparison to our findings impossible [11].

### 4.4. Complications and Postoperative Pain

Postoperative complications occurred in both groups, and the frequency was comparable (OW: 59.4% vs. PC: 54.2%; Table 3). However, the severity of complications as rated in accordance with the Clavien–Dindo classification [29] differed considerably. In the PC group, one patient suffered grade I bleeding whereas in the OW group, two (6.23%) patients experienced grade I bleeding and three (9.4%) patients had grade III bleeding. On the other hand, patients in the PC group experienced a higher rate of wound healing problems. Most of the affected patients suffered from partial wound dehiscence, which had no serious consequences. In the OW group, six of 26 (23.1%) patients discontinued VAC^®^ treatment, mainly because of severe pain that made VAC^®^ treatment no longer tolerable.

In total, six of 32 (18.7%) patients in the OW group and seven of 24 (29.2%) patients in the PC group experienced grades I and II SSIs. This was higher than the SSI rate (12.8%) reported by Al-Khayat et al. for PSD patients treated by Karydakis flap transposition [13]. However, Al-Khayat et al. mentioned that these SSIs required drainage of the wound, indicating that the grade of complication according to Clavien–Dindo classification was probably higher than in our patients [13,29].

Yildiz et al. reported a higher rate of complications (87.5%) in teenagers suffering from PSD treated with elliptical excision and median skin closure when compared to the rate of complications in patients treated with excision and Limberg flap coverage (15.6%) [8].

In our study, postoperative pain was minimal in both groups, with somewhat lower scores recorded for the PC group than the OW group on days 1 to 6 after the operation. The highest score measured was 3.2 on day 5 in the OW group. Thus, mean VAS scores remained below 4 at all time points, rendering additional pharmacologic pain management unnecessary. 

A Cochrane meta-analysis did not bring to light any differences with respect to postoperative complications and LOS [1]. This notion is corroborated by the guidelines of the ASCRS [5]. A meta-analysis of RCTs with a follow-up > 1 year revealed a similar rate of postoperative infective complications after OW and PC. OW reduced the risk of PSD recurrence by 58% when compared to excision and primary wound closure [2]. Of note, patients treated with primary wound closure were able to return to work earlier [1,14]. 

When comparing off-midline wound closure techniques to midline closure techniques, clear benefits of off-midline wound closure were noted with respect to PSD recurrence rate, postoperative infections, and other postoperative complications [1,4]. However, we agree with Humphries et al. who stated that no treatment method can eliminate the risk of recurrence [37].

### 4.5. Additional Anesthetic Procedures and Seniority of Surgeons

In the OW group, seven (21.8%) patients had to undergo one to three additional anesthetic procedures which were mostly for VAC^®^ changes in the operating room. 

In our study, LOS did not appear to depend on the seniority of the surgeon in charge of the operation (pediatric surgical resident vs. attending pediatric surgeon; Table 1). In line with our findings, Iesalnieks et al. reported that the seniority of the surgeon was not associated with PSD recurrence after surgery [14]. 

### 4.6. Economic and Social Impact of PSD

We estimated the healthcare costs for inpatient hospital stay at CHF 9000 (USD 9214). This important aspect should be borne in mind in the context of healthcare expenditures. We attribute these high hospital costs not only to the long inpatient stays, but also to the high healthcare costs in Switzerland. Iesalnicks et al. also found that excision and open wound treatment causes a prolonged postoperative healing time and socio-economic burden for the patients [14]. In the PC group, seven (58.3%) patients were able to return to school or work after 2 to 4 weeks, while in the OW group, only four (33.3%) patients required the same length of time. PSD excision and PC required an inpatient stay for 6 days.

We recommend that patients should not sit on the wound for 4 weeks and should refrain from competitive sports for 2 to 3 months. Our recommendation agrees with that proposed by Yildiz et al. for postoperative care of teenagers who underwent Limberg flap procedures [8].

### 4.7. Esthetic Considerations

**Figure 2:**
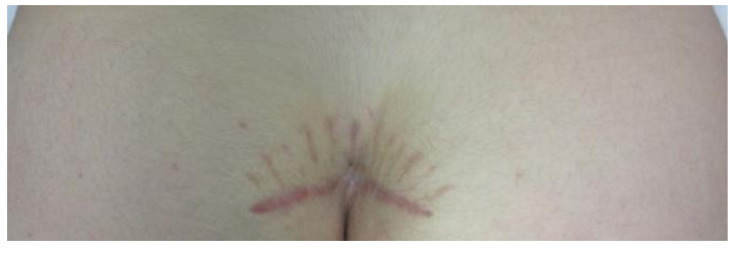
Appearance of scar 6 months after excision of pilonidal sinus disease (PSD) and primary transverse closure.

Esthetic appearance of the scar after surgical treatment of PSD represents an important factor influencing the patient’s choice of treatment. In a comparison of different surgical strategies to manage PSD in patients aged ≥14 years, Erkent et al. investigated recurrences and SF-36 score cosmetic outcomes [38]. They noted lower recurrence rates after Limberg flap repair (8%) and Karydakis flap repair (3.1%) when compared to PMC (10.8%). Despite these findings, the authors reported a preference for surgical treatment by excision and PMC among women because of cosmetic considerations [38]. Similarly, Kalaiselvan et al. stated that reconstructive techniques flattening the natal cleft may cause cosmetic issues that are deemed unacceptable by patients [39]. Thus, we hypothesize that our method of PC will not be the first choice in patients due to cosmetic concerns (Figure 2).

Midline closure results in a symmetric and inconspicuous scar, but long-term patient satisfaction hinges on their recurrence-free survival rather than cosmetic aspects [40].

### 4.8. Less Invasive PSD Treatment Options

As stated by Harris et al., there is no gold standard for the treatment for PSD, and a combination of optimized hygiene measures, removal of natal cleft hair in hirsute patients, and surgical treatment are applied at the surgeon’s discretion [41]. PSD recurrence rates after wide excision surgery may be as high as 40% [42]. Restriction of social activities, painful PSD recurrence, disruption of schooling, and delay of professional training may have psychological consequences for adolescents at this crucial time of development [43].

Thus, minimally invasive techniques to treat PSD are currently preferred because of limited scar formation and low recurrence rates associated with this approach [35,36,44,45]. However, only a few reports on such procedures in children are available [46,47].

#### 4.8.1. Early Minimally Invasive Techniques

In 1965, Lord and Millar were the first to describe a minimally invasive approach for the treatment of PSD [48]. Some years later, Bascom published a minimally invasive pit excision procedure combined with lateral incision to remove the abscess wall and remaining hair and debris [49]. This approach, known as the Bascom I procedure, is usually performed under general anesthesia and avoids excision of large amounts of tissue. In a study of day-surgical management of symptomatic PSD, only 10% of patients treated by Bascom I procedure required reoperation [50].

#### 4.8.2. Minimal but Complete Surgical Excision of PSD with Healing by Secondary Intention

Burney described the outcomes after minimal but complete surgical excision of PSD with healing by secondary intention, followed by continuous hair removal for at least 1 year [51]. In total, 570 PSD patients treated by a single surgeon were included in this retrospective survey with a mean follow-up of 4.7 years. The author emphasized that patients and caregivers must be instructed in correct wound care, including three changes of water-moistened cotton gauze a day. Patients were followed up in 10-day to 20-day intervals to ensure adequate postoperative care and proper removal of hair in the natal cleft region [51]. In total, nine of 570 (1.6%) patients experienced wound disruption after 3 to 4 months, but the wounds healed properly after repeated application of moist cotton gauze dressings. Only 3.2% of patients experienced persistent or recurrent PSD requiring reoperation. Burney concluded that that wound care, rather than the extent of excised tissue, is the most relevant aspect of successful PSD treatment [51].

#### 4.8.3. Endoscopic Pilonidal Sinus Treatment (EPSiT) 

In EPSit, a monopolar electrode inserted through the working channel of the fistuloscope is used to coagulate the wall of the pilonidal sinus cavity and shrink the fistulous tracts under direct endoscopic vision [52,53]. In children, EPSiT yielded promising results with low rates of wound infections (5.2%) and PSD recurrence (18.9%) at a follow-up interval of 11.9 months [20]. Pini Prato et al., who popularized minimally invasive EPSiT treatment of PSD, stated that PSD recurrence rates range from 20% to 30% regardless of the treatment method used [54]. Their findings compare well with ours. A first systematic review of EPSiT demonstrated high cure rates, low recurrence rates (<5%), high patient satisfaction, and little time off work or school [55]. However, in the guidelines elaborated by the ASCRS, only a weak recommendation for endoscopic and video-assisted treatment of PSD was made [4]. 

In their review comparing EPSiT to other minimally invasive PSD treatment options, Kalaiselvan et al. found no significant differences in complication rates and time to return to work or school between groups [39]. However, minimally invasive treatment strategies proved favorable over excision surgery in terms of lower complication rates, faster return to work, shortened wound healing times, and lower pain scores [39].

#### 4.8.4. Gips Minimally Invasive Treatment Technique Using Trephines

Gips minimally invasive treatment involves limited pit excision using trephines with curetting of the cavities and sinus tracts and careful removal of hairs and debris [35]. When comparing Gips sinusectomy with wide excision, Speter et al. observed no significant difference in terms of outcomes but noted a reduced number of sick days and shorter time to full activity [56]. Similar conclusions emerged from a 10-year review of the Gips technique practiced at the Israeli Defense Forces (IDF) military hospitals to treat military personnel with PSD [57]. The Gips procedure resulted in fewer sick-leave days and very low rates of PSD recurrence when compared to other surgical techniques applied in public hospitals [57].

#### 4.8.5. Minimally Invasive Pilonidal Protocol (MIPP) 

In an attempt to optimize resource utilization and outcomes, Boston Children’s Hospital implemented a minimally invasive pilonidal protocol (MIPP) in 2016. They compared the outcomes of 34 PSD patients treated according to the MIPP with those of children treated with surgical excision [46]. MIPP comprises incision and drainage of acute abscesses, daily soaking, manual hair removal until drainage has improved, and outpatient sinusectomy and debridement using local anesthesia. Any hair retained subcutaneously is removed using a clamp, and the pit openings are closed by sutures. 

Antibiotic ointment is used to cover the sutures, and daily showering is recommended. Patients have to undergo regular clinical follow-up consultations [58]. Additionally, serial postoperative laser epilation treatments are performed in hirsute patients. The MIPP protocol does not suggest any restriction of activities, sports, and schooling or work. MIPP utilization resulted in significantly higher healing rates or improvement of symptoms as well as lower hospital costs [46]. Thus, the authors recommend MIPP implementation to improve PSD treatment and lower PSD recurrence in children [46].

#### 4.8.6. Use of Fibrin Sealant after Pit Excision

In 2005, Lund and Leveson first described the successful use of fibrin glue in the management of PSD [59]. A review of the effectiveness of PSD treatment by pit excision and fibrin glue application revealed low PSD recurrence rates (1.2%) and short times to return to normal activities in adults [60].

Smith et al. published the first retrospective study in a group of children (median age 15 years) who underwent excision of the pilonidal sinus tracts and pits with a fine surgical blade, followed by curettage of the sinus tract with a cytology brush and subsequent filling of the cavity with fibrin glue [61]. When comparing this group to a group of children treated with lateralizing flap techniques, the authors noted no clear difference in primary PSD recurrence rates (20% vs. 15%) and frequency of wound infections between the groups. Wound healing time after fibrin glue application was shorter than that after traditional PSD treatment techniques [62]. Hardy et al. confirmed their promising results with fibrin glue obliteration (FGO) in children in a series of 18 adolescents (median age 16 years) and reported return to normal activities within 3 days and absence from school of only 1 day [63]. PSD recurrence was related to the number of pits. In case of recurrence, curettage and FGO treatments were repeated [63].

#### 4.8.7. Phenol Treatment of PSD

Phenolization has been shown to result in a shortened time to complete wound healing (mean 16 days) and no time off work in adults [64]. Application of crystallized phenol in children suffering from PSD should be performed under anesthesia even in the day clinic setting [9]. It must be noted that use of crystallized phenol is forbidden in some countries (e.g., Germany) due to the severe toxicity of phenol [2,19]. Nonetheless, the ASCRS issued a strong recommendation for using phenol in patients with acute or chronic PSD without abscess [4].

Sakçak et al. reported improved outcomes in terms of skin burns and fatty tissue necrosis when injecting only 1 mL of 40% phenol solution into the main sinus orifice after removing hair, debris, and infected contents from the main sinus, using a curved mosquito clamp [65]. They stated that phenol treatment is advisable only if the sinus is uncomplicated and noninfected. 

To protect the skin surrounding the sinus orifices from maceration, necrosis, and burns after phenol application, many authors recommend applying oily pomades around the sinus orifices [9]. To prevent fat tissue necrosis and cellulitis due to phenol application, meticulous hemostasis and instillation of phenol crystals or instillation of liquid phenol solution without applying pressure have been recommended [9].

#### 4.8.8. Manual Removal of Hair and Permanent Laser Hair Epilation at the Natal Cleft

In 1994, Armstrong and Barcia proposed manual hair removal at the region of the natal cleft as an effective treatment of chronic PSD [66]. Some 10 years ago, permanent laser hair epilation was first shown to reduce the rate of PSD recurrences [67,68]. In 2017, Dessiley et al. reported a new minimally invasive technique to treat PSD using diode laser, similar to the laser technique used to treat anal fistulas [69]. They described the insertion of a diode laser into sinus tracts which had been carefully debrided under local anesthesia. This technique was termed sinus laser therapy (SiLaT). Using a small incision, a radial fiber for transmission of laser light with a wavelength of 1470 nm is inserted into the sinus tracts, and laser light is delivered at continuous mode [69]. SiLaT is used to destroy granulation tissue and shrink the fistulous tracts and cavities [70]. In 2018, Pappas and Christodoulou reported a healing rate of 90.3% after single SiLaT in chronic PSD and described promising results in patients suffering from relapsing PSD [70]. Unfortunately, laser epilation treatment is generally not covered by the health insurances in many countries such as Switzerland and the USA [46,71].

At our hospital, we recommend privately financed laser hair epilation at the region of the natal cleft in hirsute children. However, only 50% of families in our study agreed to pay for the costs of a series of laser hair epilation sessions. Levinson et al. hypothesized that in soldiers with a history of PSD surgery who carry an increased risk of PSD recurrence after Gips minimal surgery trephine technique, laser hair epilation in the natal cleft region should be considered [57].

### 4.9. Current Paradigm Shift towards Minimally Invasive Techniques to Treat Chronic PSD 

Guidelines of the Italian Society of Colorectal Surgery (SICCR) published in 2015 recommend that minimally invasive techniques should be considered in the treatment of PSD [72]. Minimally invasive approaches are associated with less interference of PSD with social life and activities [36]. Therefore, we agree with Soll et al. that “less is more” in PSD [45]. Thus, a paradigm shift is required to reduce the toll of surgical PSD treatment on patients, families, and the healthcare system [46].

### 4.10. Strengths and Limitations of the Study

As various surgeons had performed the operations in this study, our study results can be generalized, which represents a strength of this investigation. Moreover, our datasets were almost complete. In contrast, the study was limited by its retrospective and nonrandomized design which did not allow uniform sample size calculation, randomization, and follow-up analyses. Another limitation may have been a certain treatment bias since the surgeon in charge chose the type of PSD treatment considered appropriate after discussing it with the patient and his/her parents. Moreover, we included a limited number of patients, i.e., the study was not powered for hypothesis testing. In addition, divergent follow-up intervals and volumes of excised tissue in the two groups may have caused bias regarding recurrence rates.

We presume that a steep learning curve occurred during the switch from OW to PC in the course of the study period. Therefore, the performance might have been suboptimal in the first few PC operations conducted.

There are more than 100 surgical techniques to treat PSD. Nonetheless, morbidity and rates of PSD relapse have changed only minimally over time, regardless of the operative technique applied [73]. We are aware that the description of a non-standard cleft lift procedure may be of limited benefit to surgeons and patients. However, in line with other authors we feel that studies on PSD outcome in children are needed since treatment modalities appear to be less favorable in children than in adults [56]. 

## 5. Conclusions

In our study, the rate of PSD recurrence proved to be markedly, albeit nonsignificantly, lower in the PC group than the OW group (12.5% vs. 37.5%; ratio: 0.19 (95% CI, 0.03–1.07)). In addition, there were no high-grade complications in the PC group, and postoperative pain was minimal. 

To neutralize confounding, e.g., by selection bias, RCTs are required to confirm our findings. Moreover, adequately powered, prospective studies are needed to compare PSD recurrence rates after primary transverse closure with those associated with the favored minimally invasive techniques as well as the well-established asymmetric excision techniques with lateralization of the scar in children.

Less invasive treatment approaches to treat chronic PSD are typically performed in an outpatient setting and offer reduced morbidity, low recurrence rates, and shortened periods of time to return to work or social activities. In conclusion, more radical operations of PSD should be reserved for recurrent PSD where less invasive approaches have failed several times.

## Figures and Tables

**Table 1:** Patient characteristics and surgical details.

	Open Wound Care (OW)	Primary Transverse Closure (PC)	*p*
*n*	32	24	
Sex = m (%)	19 (59.4)	17 (70.8)	0.546
Age (years; mean (SD))	15.5 (1.0)	15.5 (1.4)	0.938
Previous abscess incision (%)	17 (53.1)	8 (33.3)	0.192
Weight percentile (%)			0.601
<3	0 (0.0)	1 (4.2)	
10–25	1 (3.1)	1 (4.2)	
25–50	1 (3.1)	2 (8.3)	
50–75	4 (12.5)	2 (8.3)	
75–90	5 (15.6)	6 (25.0)	
90–97	5 (15.6)	5 (20.8)	
>97	16 (50.0)	7 (29.2)	
Surgical technique (%)			<0.001
Open	6 (18.8)	0 (0.0)	
VAC^®^	26 (81.2)	0 (0.0)	
Transverse	0 (0.0)	24 (100.0)	
Surgeon = attending surgeon (%)	11 (34.4)	12 (50.0)	0.367
Volume specimen (cm^3^; mean (SD)) *	28.7 (37.9)	19.2 (22.0)	0.284
Number of VAC^®^ changes (%)			<0.001
0	7 (21.9)	24 (100.0)	
2	3 (9.4)	0 (0.0)	
3	1 (3.1)	0 (0.0)	
4	5 (15.6)	0 (0.0)	
6	3 (9.4)	0 (0.0)	
7	1 (3.1)	0 (0.0)	
8	4 (12.5)	0 (0.0)	
9	2 (6.2)	0 (0.0)	
10	1 (3.1)	0 (0.0)	
12	2 (6.2)	0 (0.0)	
13	2 (6.2)	0 (0.0)	
14	1 (3.1)	0 (0.0)	

* In two patients (*n* = 2), the volume of excised specimen was not recorded.

**Table 2:** Inpatient LOS, outpatient LOS, total LOS, and duration of absence from school or work.

	Open Wound Care (OW)	Primary Transverse Closure (PC)
*n*	32	24
Duration outpatient stay in days (median [IQR])	8.5 [5.5, 14.5]	3.0 [2.0, 4.8]
Duration inpatient stay in days (median [IQR])	6.5 [3.8, 12.0]	6.0 [5.0, 7.0]
Overall LOS (median [IQR])	15.8 [10.2, 25.5]	9.0 [7.5, 11.5]
Time off from school or work (%) *		
0–2 weeks	3 (25.0)	1 (8.3)
2–4 weeks	4 (33.3)	7 (58.3)
4–6 weeks	2 (16.7)	1 (8.3)
6–8 weeks	2 (16.7)	1 (8.3)
12–14 weeks	1 (8.3)	1 (8.3)
>14 weeks	0 (0.0)	1 (8.3)

* Available for only 12 patients in each group (37.5% in OW group and 50.0% in PC group); IQR: interquartile range; LOS: length of hospital stay.

**Table 3:** Type, grade, and rate of complications categorized according to the Clavien–Dindo classification of surgical complications (grades I to III).

	Open Wound Care (OW)	Primary Transverse Closure (PC)
*n*	32	24
Complications (%)	19 (59.4)	13 (54.2)
Bleeding (%)		
None	27 (84.4)	23 (95.8)
Grade I	2 (6.2)	1 (4.2)
Grade III	3 (9.4)	0 (0.0)
SSI (%)		
None	26 (81.2)	17 (70.8)
Grade I	1 (3.1)	1 (4.2)
Grade II	5 (15.6)	6 (25.0)
Wound healing disorders (%)		
None	25 (78.1)	13 (54.2)
Grade I	5 (15.6)	11 (45.8)
Grade II	2 (6.2)	0 (0.0)
Complications with VAC^®^ (%)		
None	24 (75.0)	24 (100.0)
Grade I	1 (3.1)	0 (0.0)
Grade II	1 (3.1)	0 (0.0)
Grade III	6 (18.8)	0 (0.0)
Allergies (%)	1 (3.1)	2 (8.3)

SSI: surgical site infection.

**Table 4:** Change of mean visual analog scale (VAS) scores from the day of operation (OW or PC) up to postoperative day 6.

	Mean VAS Score OW Group	Mean VAS Score PC Group
Day of operation	1.2	1.4
Postoperative day 1	1.3	1.1
Postoperative day 2	1.7	0.9
Postoperative day 3	2.2	1.0
Postoperative day 4	2.5	0.8
Postoperative day 5	3.2	0.0
Postoperative day 6	2.1	0.0

OW: open wound care; PC: primary transverse closure.

**Table 5:** Overall pilonidal sinus disease (PSD) recurrence rates, number of laser treatments, and additional anesthesia and surgeries.

	Open Wound Care (OW)	Primary Transverse Closure (PC)
*n*	32	24
Recurrence (%)	12 (37.5)	3 (12.5)
Surgeries in total (%)		
1	20 (62.5)	21 (87.5)
2	7 (21.9)	1 (4.2)
3	3 (9.4)	1 (4.2)
4	1 (3.1)	0 (0.0)
5	1 (3.1)	1 (4.2)
Additional anesthesia (%)		
0	25 (78.1)	24 (100.0)
1	5 (15.6)	0 (0.0)
3	2 (6.2)	0 (0.0)
Laser (%)	17 (53.1)	12 (50.0)
Number of laser treatments (%)		
0	16 (50.0)	12 (50.0)
2	2 (6.2)	2 (8.3)
3	1 (3.1)	6 (25.0)
4	2 (6.2)	0 (0.0)
5	1 (3.1)	1 (4.2)
6	1 (3.1)	0 (0.0)
7	4 (12.5)	1 (4.2)
9	1 (3.1)	1 (4.2)
10	3 (9.4)	0 (0.0)
11	1 (3.1)	0 (0.0)
12	0 (0.0)	1 (4.2)
